# Disruption of the Physical Interaction Between Carbonic Anhydrase IX and the Monocarboxylate Transporter 4 Impacts Lactate Transport in Breast Cancer Cells

**DOI:** 10.3390/ijms252211994

**Published:** 2024-11-08

**Authors:** Jacob E. Combs, Akilah B. Murray, Carrie L. Lomelino, Mam Y. Mboge, Mario Mietzsch, Nicole A. Horenstein, Susan C. Frost, Robert McKenna, Holger M. Becker

**Affiliations:** 1 Department of Biochemistry and Molecular Biology, University of Florida, Gainesville, FL 32611, USAmario.mietzsch@ufl.edu (M.M.); sfrost@ufl.edu (S.C.F.); 2 Department of Chemistry, University of Florida, Gainesville, FL 32611, USA; horen@chem.ufl.edu; 3 Institute of Physiological Chemistry, University of Veterinary Medicine Hannover, 30559 Hannover, Germany; 4 Department of Gastroenterology, Hepatology, Infectious Diseases and Endocrinology, Hannover Medical School, 30625 Hannover, Germany

**Keywords:** BGal2C, breast cancer, carbonic anhydrase IX, drug development, MCT1/4, protein–drug interaction

## Abstract

It has been previously established that breast cancer cells exhibit high expression of the monocarboxylate (lactate) transporters (MCT1 and/or MCT4) and carbonic anhydrase IX (CAIX) and form a functional metabolon for proton-coupled lactate export, thereby stabilizing intracellular pH. CD147 is the MCT accessory protein that facilitates the creation of the MCT/CAIX complex. This study describes how the small molecule Beta-Galactose 2C (BGal2C) blocks the physical and functional interaction between CAIX and either MCT1 or MCT4 in Xenopus oocytes, which reduces the rate of proton and lactate flux with an IC_50_ of ~90 nM. This value is similar to the K_i_ for inhibition of CAIX activity. Furthermore, it is shown that BGal2C blocks hypoxia-induced lactate transport in MDA-MB-231 and MCF-7 breast cancer cells, both of which express CAIX. As in oocytes, BGal2C interferes with the physical interaction between CAIX and MCTs in both cell types. Finally, X-ray crystallographic studies highlight unique interactions between BGal2C and a CAIX-mimic that are not observed within the CAII active site and which may underlie the strong specificity of BGal2C for CAIX. These studies demonstrate the utility of a novel sulfonamide in interfering with elevated proton and lactate flux, a hallmark of many solid tumors.

## 1. Introduction

Breast cancer is the most commonly diagnosed form of cancer and the leading cause of cancer death in women worldwide, with over two million new cases and over half million fatalities in 2022 [1]. There are three major subgroups of breast cancer that can be further subdivided based on gene expression profiles [2]. These include tumors that express hormone receptors for estrogen (ER) and/or progesterone (PR) (the luminal phenotype), which include A and B subtypes, the latter of which predicts less positive outcomes; those that express HER2 (ERBB2) receptors; and those that express neither the hormone nor HER2 receptors (the “triple negative” (TNBC) phenotype), which is one of the most aggressive forms of breast cancer, exhibiting the highest mortality rates [3]. TNBC tumors are extremely invasive, leading to high rates of recurrence post treatment and low recurrence-free survival [4,5,6]. Approximately 70% of breast cancers are ER/PR positive, 15% are HER2+, and 15% have the TNBC phenotype, of which the latter contains most of the BRCA1 mutations [3].

Hypoxia typifies the environments of many aggressive breast cancers, which upregulates the expression of the constitutive glucose transporter (GLUT1) and selects steps in glycolysis and upregulation of the monocarboxylate (lactate) transporter MCT4 [7,8,9,10]. This shifts the dependency of tumors from oxidation phosphorylation for energy production to glycolysis, a process known as the “Warburg Effect” [11]. Export of lactate and protons is required both to prevent intracellular acidification and cell death [12] and also create an acidic microenvironment, to which cancer cells adapt [13] and that favors their survival [14,15]. Indeed, while normal tissues display extracellular pH values of ~7.3 to 7.4 and extracellular lactate concentrations of 1.5 to 3 mM, extracellular pH can drop down to values as low as 6.5 in tumor tissue, while lactate concentrations can rise to levels up 10 to 30 mM [16,17,18]. This creates a toxic environment for the surrounding normal cells [19,20] and dampens immune response [21].

MCT4 and MCT1 are primarily utilized to shuttle lactate and protons out of cancer cells [9,22,23,24], which provides a survival advantage. MCT1 has a K_m_ value of 3–5 mM for lactate [25,26], while MCT4 displays a K_m_ value of 17–35 mM [27], which renders the two transporters ideal for lactate shuttling under physiological and pathophysiological conditions.

CD147 serves as a chaperone for the MCTs, which drives their localization to the plasma membrane and regulates catalytic activity [28,29]. CD147 is upregulated in TNBC [24] and is correlated with increasing histological grade [30,31] and associated with shorter progression-free survival chemoresistance [30,32].

In addition to upregulation of glycolysis and the MCTs, hypoxia also increases the expression of carbonic anhydrase IX (CAIX) [33]. CAIX is a membrane-bound form of the carbonic anhydrase (CA) family of isozymes that catalyze the interconversion of carbon dioxide (CO_2_) and water to bicarbonate (HCO_3_^−^) and a proton (H^+^) [34,35]. CAIX expression is associated with aggressive breast cancers, where it plays a role in pH control of the tumor microenvironment [15,36,37]. In addition, CAIX has been shown to form a protein complex, coined “transport metabolon”, with MCTs in breast cancer cells to facilitate lactate export under hypoxic conditions [12,38,39]. Within this complex, the CAIX catalytic domain binds to the extracellular domain of CD147, which brings CAIX close to the transporter [12]. Interestingly, binding to CD147 is mediated by CAIX-His200, which does also function as an intramolecular proton-shuttle for the enzyme [12]. Closely positioned to the transporter, the CAIX proteoglycan-like domain can function as a proton antenna, which rapidly shuttles protons between the MCT transporter pore and surrounding protonatable residues at the cell surface to speed up proton-coupled lactate transport across the cell membrane [39]. A previous study was able to demonstrate that disruption of the transport metabolon with an antibody, targeted against the CAIX-binding site in CD147, reduces proton-coupled lactate transport and inhibits proliferation of breast cancer cells under hypoxia [12]. However, we assume that CAIX, which is overexpressed in many cancers but only rarely found in healthy tissue, poses a better target for metabolon disruption than the ubiquitously expressed CD147. Therefore, we were aiming for the development of a CAIX-specific inhibitor that could target the direct interaction between CAIX and the MCT-CD147 complex.

One of the important issues in the development of such drugs is their specificity for CAIX over the other members of this family, particularly CAII, which is the abundant and widely expressed cytosolic form of the CA family. Acetazolamide is the classical inhibitor of CAII [40] but functions equally so for several other CA family members [41]. The sulfonamide structure, however, has been exploited to create a variety of inhibitors that select for CAIX over CAII [42]. We and others have developed compounds that take advantage of the region outside of the zinc-containing catalytic pocket, which in CAIX differs in amino acid composition from CAII and is known as the “selective pocket”, which also provides specificity for binding to CAIX [38,39,43,44]. We have recently exploited the finding that sugar moieties in sulfonamide-based CA inhibitors provide selectivity for CAIX [45] and that saccharin (SAC) shows strong specificity for CAIX over CAII [46]. As such, we have developed a new technique for generating derivatives of SAC containing glucose or galactose “tails” [47], one of which is selective toward CAIX, which is used in the present study: Beta-Galactose-2C (BGal2C). We now test whether BGal2C can target the MCT-CAIX transport metabolon to prevent the CAIX-mediated increase in lactate flux observed in hypoxic breast cancer cells.

## 2. Results

### 2.1. BGal2C Prevents CAIX-Mediated Increase in Proton and Lactate Flux in Xenopus Oocytes

**Figure 1:**
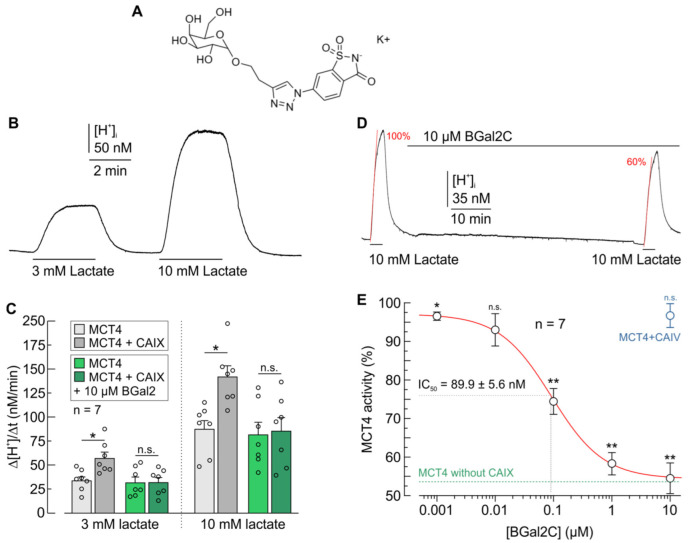
BGal2C inhibits the CAIX-induced increase in proton flux in *Xenopus* oocytes. (**A**) Chemical structure of BGal2C. (**B**) Example of an original recording of the intracellular H^+^ concentration in MCT4 + CAIX-expressing *Xenopus* oocytes. (**C**) Rate of change in intracellular H^+^ concentration (Δ[H^+^]/Δt) during application of 3 and 10 mM lactate in oocytes expressing MCT4 (lighter shaded bars) or MCT4 + CAIX (darker shaded bars). Gray bars represent control cells; green bars represent cells incubated with 10 μM BGal2C for 1 h. * *p* ≤ 0.05, n.s. not significant; ANOVA; Mean + S.E.M. (**D**) Original recording of intracellular H^+^ concentration in a MCT4 + CAIX-expressing oocyte. (**E**) Relative MCT4 transport activity is plotted against the BGal2C concentration. The regression curve (red line) was created using a Hill1 fit. The green dotted line indicates relative MCT4 transport in oocytes expressing MCT4 without CAIX. The blue data point shows the relative MCT4 transport activity in MCT4 + CAIV-expressing oocytes in the presence of 10 μM BGal2C. * *p* ≤ 0.05, ** *p* ≤ 0.01; paired *t*-test; mean ± S.E.M.

The structural formula of BGal2C is shown in Figure 1A. The synthesis of the inhibitor was described in a previous publication [47]. As reported earlier, the transport activity of MCTs is facilitated by direct interaction with CAIX [48,49]. This interaction is independent from carbonic anhydrase catalytic activity, but it requires direct binding of CAIX to the transporter’s chaperon CD147 [12]. This binding has been shown to be mediated by the CAIX-His200 proton shuttling residue, which is located close to the enzyme’s catalytic pocket [12]. To test whether BGal2C can inhibit CAIX-mediated increase in MCT transport activity, we coexpressed MCT4 with CAIX in Xenopus oocytes. Because Xenopus oocytes express an endogenous analogue of CD147 [50], heterologous coexpression of the chaperon is not required [12,51]. Transport activity of the H^+^/lactate cotransporter MCT4 was determined by monitoring the change in intracellular H^+^ concentration during application of 3 and 10 mM lactate (Figure 1B). Previous studies have shown that these two lactate concentrations give optimal results for the study of MCT transport function on Xenopus oocytes [52,53,54]. Coexpression of MCT4 with CAIX resulted in a significant increase in MCT4 transport activity, as measured by the rate of rise in intracellular H^+^ concentration (ΔH^+^/Δt) during lactate application (Figure 1C; gray bars). Pre-incubation of MCT4 + CAIX coexpressing oocytes with 10 µM of BGal2C resulted in complete inhibition of the CAIX-mediated increase in transport activity (Figure 1C; green bars). However, activity of MCT4 was not directly affected by BGal2C in the absence of CAIX (Figure 1C). Western blot analysis confirmed that the BGal2C-induced decrease in MCT4 transport activity was not due to a reduced expression of MCT4 in the oocytes (Figure S1).

To determine the inhibition constant (IC_50_) of BGal2C for the CAIX-mediated increase in MCT4 transport activity, we generated a dose response curve in Xenopus oocytes. MCT4 transport activity was determined by measuring the rate of change in intracellular H^+^ concentration (ΔH^+^/Δt) during application of 10 mM lactate before and after a 1 h application of BGal2C (Figure 1D). The experiment was repeated with a range of BGal2C concentrations from 1 nM to 10 µM. Regression analysis revealed an IC_50_ of 89.9 ± 5.6 nM for BGal2C for the CAIX-mediated increase in MCT4 activity (Figure 1E).

The Isoform-specificity of BGal2C was checked in Xenopus oocytes that coexpressed MCT4 and CAIV. CAIV, an isozyme of the carbonic anhydrase family, which is attached to membranes via a glycosylphosphatidylinositol (GPI) tail, has been previously shown to facilitate MCT transport activity in a similar fashion as CAIX [51,53]. Coexpression of CAIV increased MCT4 transport activity to a similar extent as CAIX did (from 56.1 ± 7.9 to 80.4 ± 6.2 nM/min; *p* < 0.05, *n* = 7). However, application of 10 µM BGal2C had no significant effect on the CAIV-mediated increase in MCT4 activity (Figure 1E, blue data point).

### 2.2. BGal2C Blocks CAIX-Mediated Enhancement of MCT-Dependent Lactate Flux in Breast Cancer Cells

**Figure 2:**
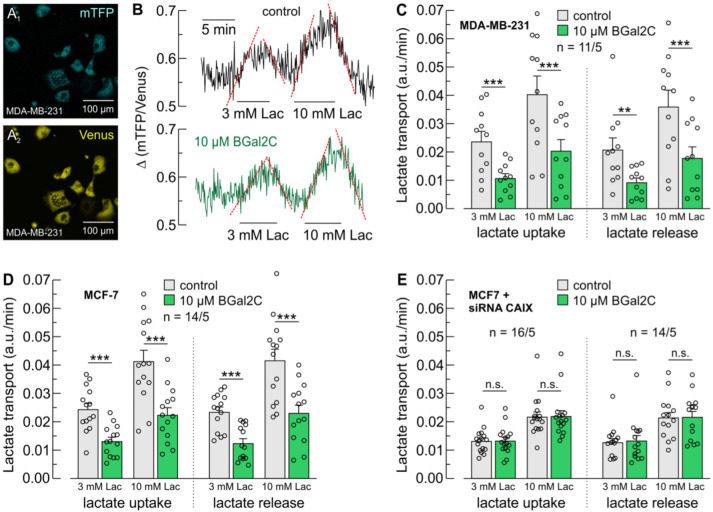
BGal2C inhibits lactate transport in hypoxic breast cancer cells. (**A**) Fluorescent signals for mTFP (460–500 nm) ((**A_1_**), blue) and Venus (520–550 nm) ((**A_2_**), yellow) from MDA-MB-231 cells, transfected with the lactate-sensitive FRET nanosensor Laconic. (**B**) Original recordings of the relative change in intracellular lactate concentration during application of 3 and 10 mM lactate in hypoxic MDA-MB-231 cells before (black trace) and after incubation with 10 µM BGal2C (green trace). The red lines indicate the rate of change in intracellular lactate concentration (lactate transport), as shown in figure (**C**). (**C**) Rate of change in intracellular lactate concentration during application and removal of lactate in hypoxic MDA-MB-231 cells in the absence (gray bars) or presence (green bars) of 10 μM BGal2C. (**D**) Rate of change in intracellular lactate concentration during application and removal of lactate in hypoxic MCF-7 cells in the absence or presence of 10 μM BGal2C. (**E**) Rate of change in intracellular lactate concentration during application and removal of lactate in hypoxic MCF-7 cells, in which CAIX was knocked down with siRNA, in the absence or presence of 10 μM BGal2C. ** *p* ≤ 0.01, *** *p* ≤ 0.001, n.s. not significant; paired *t*-test; mean ± S.E.M.; *n* = number of cells/number of batches.

**Figure 3:**
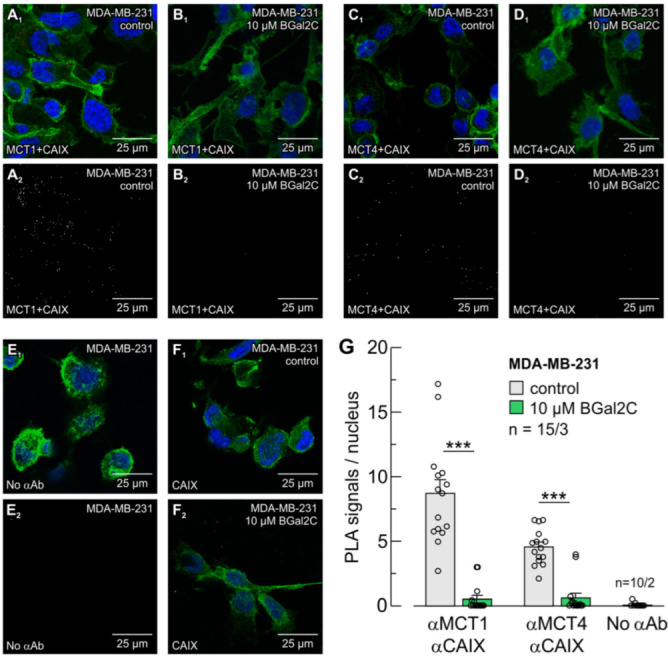
BGal2C disrupts the transport metabolon formed between MCTs and CAIX in MDA-MB-231 cells. In situ proximity ligation assay (PLA) for MCT1 + CAIX (**A**,**B**) and MCT4 + CAIX (**C**,**D**) in hypoxic MDA-MB-231 cells. Primary antibodies (α) included those for MCT1, MCT4 (as appropriate) and CAIX. Cells were pre-incubated without (**A**,**C**) or with 10 μM BGal2C for one hour (**B**,**D**). For controls, the PLA was performed without primary antibodies (**E**). Panels (**A_1_**–**E_1_**) show the PLA signals (red), nuclei staining (blue) and actin staining (green). Panels (**A_2_**–**E_2_**) show exclusively the PLA signals. (**F**) Antibody staining against CAIX (green) without (**F_1_**) or with 10 μM BGal2C (**F_2_**). (**G**) Quantification of the PLA signals, as illustrated in Panels (**A**–**E**). *** *p* ≤ 0.001; ANOVA; mean + S.E.M.; *n* = number of pictures/number of batches.

MDA-MB-231 cells are representative TNBC cells and strongly express CAIX under hypoxic conditions [12,55,56]. Lactate flux in MDA-MB-231 cells is increased under hypoxia as compared to normoxic conditions [48]. The facilitation of lactate transport capacity is mediated by the non-catalytic action of CAIX, since knockdown of CAIX, but not inhibition of CA catalytic activity, abolished the hypoxia-induced increase in lactate flux [48]. To determine the effect of BGal2C on lactate transport in hypoxic MDA-MB-231 cells, we measured changes in intracellular lactate concentration during application and withdrawal of lactate with the lactate-sensitive FRET nanosensor Laconic, as previously described [48,57] (Figure 2A,B). Lactate was applied at a concentration of 3 and 10 mM before and 1 h after incubation of cells with BGal2C. The incubation was carried out under the microscope to allow measurement of lactate flux before and after application of inhibitor in the same cells. However, the measurement was stopped during that time to avoid bleaching of the FRET sensor. Application of 10 µM BGal2C resulted in a significant decrease both in lactate uptake and release (Figure 3C). These data demonstrate that BGal2C blocks both uptake and release of lactate in hypoxic MDA-MB-231 cells.

The results were confirmed by additional measurements on MCF7 cells. MCF-7 cells arose from an ER-positive breast carcinoma [58] and have luminal origins [59]. As in MDA-MB-231 cells, lactate flux in MCF7 cells was shown to increase under hypoxia, mediated by the non-catalytic action of CAIX [12,48]. Lactate transport was again determined by measurement of changes in intracellular lactate concentration during application and withdrawal of lactate with Laconic. Application of 10 µM BGal2C for 1 h again resulted in a significant decrease in lactate uptake and release (Figure 2D,E). Interestingly, MDA-MB-231 and MCF-7 cells displayed quite similar lactate transport rates under hypoxia, even though the two cell lines have been reported to have marked differences in their energy metabolism.

To confirm that the effect of BGal2C on lactate transport is CAIX-dependent, the measurement was repeated in hypoxic MCF7 cells, in which CAIX was knocked down using siRNA. The knockdown efficiency was 98% at the RNA level. Knockdown of CAIX resulted in a significant decrease in lactate flux as compared to untreated cells (Figure 2D,E, gray bars). Furthermore, application of BGal2C did not further decrease lactate influx or efflux in CAIX knockdown cells (Figure 2D,E, green bars). These results demonstrate that BGal2C blocks the CAIX-mediated increase in lactate transport capacity in hypoxic cancer cells.

### 2.3. BGal2C Disrupts the Direct Interaction Between MCTs and CAIX

CAIX-mediated facilitation of MCT transport activity requires close colocalization between the two proteins, which is achieved by binding of CAIX to the Glu73 in the Ig1 domain of the transporter’s ancillary protein CD147 [12]. In CAIX, the binding site is His200 [12], which is also the central part of the enzyme’s intramolecular H^+^ shuttle [60]. By this binding, MCT, CD147 and CAIX form a “transport metabolon” that can drive lactate flux through the membrane. To investigate whether BGal2 disrupts the MCT-CAIX transport metabolon, we investigated the colocalization of MCTs and CAIX in hypoxic cancer cells using an in situ proximity ligation assay. In hypoxic MDA-MB-231 cells, the assay produced robust signals for MCT1 and CAIX and for MCT4 and CAIX, which demonstrates that CAIX is closely colocalized to both transporter isoforms with a maximum distance of 30–40 nm (Figure 3A,C). Pre-incubation of hypoxic cells with 10 µM of BGal2C for 1 h resulted in a significant reduction of the number of PLA signals (Figure 3B,D). The number of PLA signals for MCT1 and CAIX decreased from 8.7 ± 1.0 signals/nucleus to 0.5 ± 0.3 signals/nucleus. For MCT4 and CAIX, the signals decreased from 4.6 ± 0.4 to 0.6 ± 0.3 signals/nucleus (Figure 3E). Without a primary antibody, the assay produced only 0.1 ± 0.1 signals/nucleus (Figure 3E,G). Application of BGal2C did not alter conventional antibody staining of CAIX, which demonstrates that the inhibitor does not interfere with binding of the primary antibody (Figure 3F). These data indicate that BGal2C can block colocalization of MCT1/4 and CAIX, presumably by disruption of the transport metabolon.

**Figure 4:**
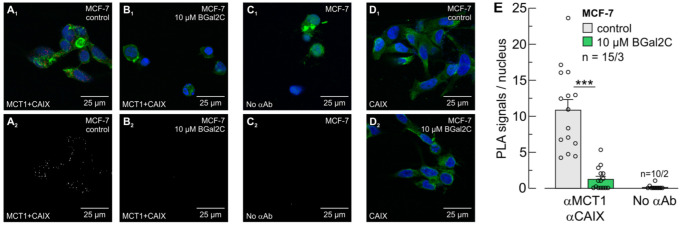
BGal2C disrupts the transport metabolon formed between MCT1 and CAIX in MCF-7 cells. In situ proximity ligation assay (PLA) for MCT1 + CAIX (**A**,**B**) in MCF-7 cells. Primary antibodies (α) included those for MCT1 and CAIX. Cells were pre-incubated without (**A**) or with 10 μM BGal2C for 1 h (**B**). For control, the PLA was performed without primary antibodies (**C**). Panels (**A_1_**–**C_1_**) show the PLA signals (red), nuclei staining (blue) and actin staining (green). Panels (**A_2_**–**C_2_**) show exclusively the PLA signals. (**D**) Antibody staining against CAIX (green) in the absence (**D_1_**) and presence of BGal2C (**D_2_**). (**E**) Quantification of the PLA signals as PLA signals per nucleus in MCF-7 cells, as described in Panels (**A**–**C**). *** *p* ≤ 0.001; ANOVA; mean + S.E.M.; *n* = number of pictures/number of batches.

The results were again confirmed with MCF7 cells (Figure 4). The MCF7 cells used in the present study (DSMZ-No. ACC 115) do not express MCT4 under hypoxia [48]. Therefore, the PLA was carried out only for MCT1 and CAIX. Pre-incubation of hypoxic cells with 10 µM of BGal2C for 1h decreased the number of PLA signals from 10.9 ± 1.5 to 1.3 ± 0.4 signals/nucleus (Figure 4A,B,E). Without a primary antibody, the assay produced only 0.1 ± 0.1 signals/nucleus (Figure 4C,E). Application of BGal2C again did not alter conventional antibody staining of CAIX (Figure 4D).

Taken together, these results indicate that BGal2C can inhibit CAIX-mediated facilitation of lactate transport via MCTs in hypoxic cancer cells by disruption of the transport metabolon.

### 2.4. BGal2c Binds Directly to the Catalytic Zinc in CAIX

**Figure 5:**
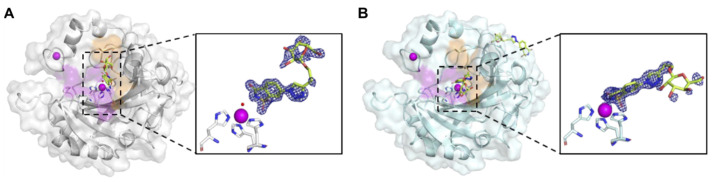
X-ray crystal structure of BGal2C bound to CAII and CAIX. The images depict a surface view of CAII (**A**) (PDB: 7RRE) and CAIX-mimic (**B**) (PDB: 7RRF), overlaid onto a cartoon backbone with the inhibitor (yellow) bound. The hydrophobic pocket (orange) and hydrophilic pocket (purple) are shaded, as are the two zinc atoms (magenta spheres). BGal2C binds through a zinc-bound solvent (red sphere) within the active site of hCAII but directly to the zinc within the active site of hCAIX-mimic in addition to on the surface. The boxes show a zoomed-in view with H94, H96 and H119 depicted as sticks. The blue mesh is the density carved to 1.5 Å with a signal to noise of 1.0.

To investigate how BGal2C disrupts the binding between CAIX and CD147, the inhibitor was soaked in CAII (PDB: 7RRE) and CAIX-mimic (a CAII scaffold, with an engineered CAIX active site, PDB: 7RRF). Of note, BGal2C bound through a zinc-bound solvent molecule in CAII (Figure 5A). The oxygen, bound to a carbon in the sulfur ring of BGal2C, forms a hydrogen bond with the nitrogen backbone of Thr199, the nitrogen backbone of Thr200 and the oxygen of Thr200. This orients the sulfur, bonded with oxygen and nitrogen, towards the zinc solvent. The middle nitrogen of the third ring in BGal2C forms a hydrogen bond with the nitrogen of Gln92. An adjoining nitrogen within the ring forms a hydrogen bond with a water molecule within the active site. Additionally, intermolecular interactions are observed with Ile91, His94, Val121, Phe131, Gly132, Val135, Leu141, Val143, Leu198 and Pro202.

The BGal2C was also crystalized with CAIX-mimic crystals (PDB: 7RRF). Interestingly, different to the CAII in complex with BGal2C, the BGal2C in CAIX-mimic bound directly to the zinc (Figure 5B). The nitrogen and oxygen atoms within the ring of BGal2C were oriented towards the zinc atom while the oxygen on the side chain of the ring was directed toward ¬histidine. The nitrogen in the sulfur ring of BGal2C formed a coordinated bond with the zinc. Oxygen bound to the sulfur in the BGal2C ring forms a hydrogen bond with the nitrogen backbone of Thr199. Oxygen bound to a carbon in the sulfur ring of BGal2C forms a hydrogen bond with a water molecule within the active site of CAIX-mimic. A side chain oxygen atom in the galactose ring forms a hydrogen bond with a water molecule, while a separate oxygen atom on a side chain of the galactose ring forms a hydrogen bond with the nitrogen of Gln92, and a third oxygen side chain on the galactose ring forms a hydrogen bond with the nitrogen of Gln67. Additionally, van der Waals interactions are observed with Leu91, Val121, Val131, Val135, Leu141, Val143, Leu198, Thr200 and Trp209 (Table S2). The refinement statistics for both structures are given in Table S1, and those for the BGal2C active site are given in Table S2.

### 2.5. Molecular Model of CAIX in Complex with CD147 with and Without BGal2c Bound

**Figure 6:**
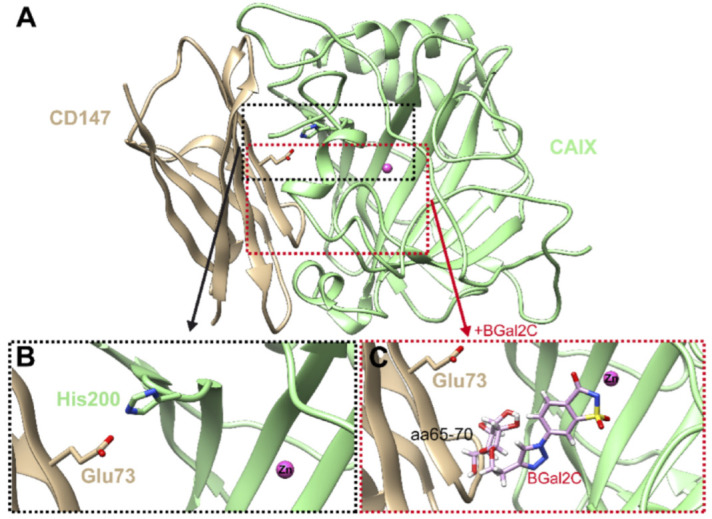
Model of the CAIX-CD147 binding interface. (**A**) Ig1 domain of CD147 (tan) and catalytic domain of CAIX (green) (taken from (12). (**B**) Interaction between CAIX His64 and CD147 Glu73. Note the distance between CD147-Glu73 and CAIX-His200 in the “in” confirmation is within hydrogen-bonding range. (**C**) The structure of BGal2C is superposed onto the CAIX-CD147 structure. Positioning of BGal2C is based on the data shown in Figure 5. Note that amino acids 65–70 of CD147 sterically clash with the sugar motif of BGal2C.

The Ig1 domain of CD147 and catalytic domain of CAIX were docked and modeled as previously described [12]. Then, the crystal structure coordinates of the CAIX-mimic complexed with BGal2c were superposed onto the CAIX structure, as described by Ames et al. [12], with a Cα RMSD of less than 1 Å, and the BGal2c coordinates were transferred into the Ames et al. model (Figure 6A). Figure 6B shows the interaction between CD147-Glu73 and CAIX-His200. From this model, it was clear that BGal2C, if bound in the CAIX catalytic domain, would sterically hinder interactions of 600 Å^2^ (ΔG −14 kcal/mol) with the Ig1 domain of CD147, especially interfering with residues 65–70 (Figure 6C). This interference would most likely also prevent the interaction between CD147-Glu73 and CAIX-His200.

## 3. Discussion

**Figure 7:**
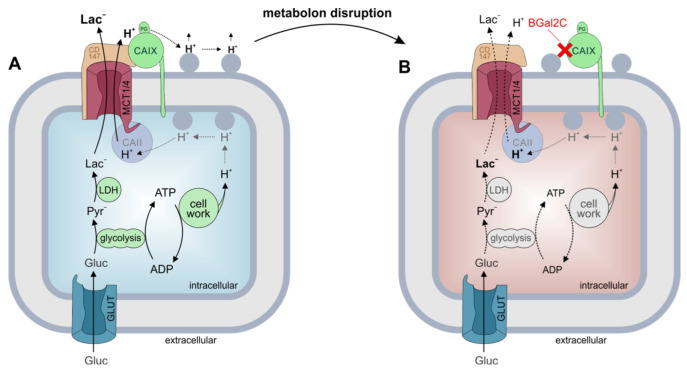
Model of the mode of action of BGal2C. (**A**) In hypoxic breast cancer cells, glycolysis is the prime energy source, which leads to vast production of lactate and protons. Both ions are rapidly removed from the cell by the transport metabolon formed between MCT and CAIX. In this complex, CAIX, which is directly bound to the Ig1 domain of the MCT1/4 chaperone CD147, serves as a proton antenna for the transporter, which rapidly exchanges H^+^ between the transporter pore and surrounding protonatable residues (blue circles) at the extracellular site of the plasma membrane. Thereby, CAIX counteracts the formation of proton microdomains around the transporter pore and drives the efflux of lactate and protons from the cell. (**B**) Binding of BGal2C to CAIX disrupts the direct interaction between CAIX and CD147. The disruption of the transport metabolon inhibits MCT transport activity, which leads to intracellular accumulation of lactate and protons, which in turn could result in a decrease in glycolytic activity and ultimately a reduction in cell proliferation.

We have previously shown that CAIX facilitates lactate export via MCT1 and MCT4 in hypoxic breast cancer cells, presumably by functioning as a “proton antenna” that mediates rapid shuttling of H^+^ to/from the transporter pore [12,48,49,61]. Facilitation of MCT transport activity is independent from CAIX catalytic activity but requires close colocalization between MCT and CAIX, which is achieved by direct binding of the enzyme to the transporter’s ancillary protein CD147 [12,48]. Since binding of CAIX to the MCT-CD147 complex is mandatory for the facilitation of proton-coupled lactate flux, we speculated that this interaction could be a target for small molecule drug design. Here, we show that the CAIX-mediated enhancement MCT4 transport activity was completely blocked by addition of BGal2C in *Xenopus* oocytes. The IC_50_ for this effect was in the low nM range (89.9 nM), which is close to the IC_50_ for inhibition of purified CAIX (93 nM; Ref. [47]). However, BGal2C did not directly inhibit MCT transport activity in the absence of CAIX, which indicates that the inhibitor specifically targets the MCT-CAIX transport metabolon while leaving lactate transporters without CAIX unaffected. This is noteworthy since MCT-mediated lactate transport plays a pivotal function in the energy metabolism of a wide range of tissues, including brain, liver and muscle [62,63,64]. Therefore, systemic application of MCT inhibitors for tumor therapy could lead to severe side effects in uninvolved tissues. Expression of CAIX, however, is restricted to a few healthy tissues, like the stomach and gallbladder, but is highly upregulated in tumor cells [65]. Indeed, we were able recently to show that MCT1 and 4 form transport metabolons with CAIX in human breast cancer tissue but not in adjacent healthy tissue [12]. In the present study, we were able to show that BGal2C inhibited both lactate uptake and release in the breast cancer cell lines MDA-MB-231 and MCF-7 under hypoxia only in the presence of CAIX. We also demonstrated, using a proximity ligation assay, that BGal2C interferes with the physical interaction between MCTs and CAIX in these cell lines. Even though BGal2C binds to CAIX at the catalytic zinc, our modeling suggests that BGal2C sterically interferes with the interaction of His200 with CD 147, especially in the region of residues 65–70. This in turn disrupts the MCT1/4-CAIX metabolon, leading to decreased MCT activity, as illustrated in Figure 7. This is the most likely cause of the accumulation of intracellular lactate and protons, which will ultimately lead to cell death.

Indeed, we were able previously to show that incubation of MDA-MB-231 and MCF-7 cells with an anti-CD147 antibody resulted in similar effects as incubation with BGal2C [12]. This antibody targets a region close to Glu73 of the IgG domain of CD147, which serves as the binding site for His200 in CAIX [12]. Application of the antibody not only displaced CAIX from the MCTs and inhibited lactate transport in hypoxic MDA-MB-231 and MCF-7 cells but resulted in decreased lactate production and restricted cancer cell growth [12].

In conclusion, this study provides proof of concept that cancer cell metabolism can be targeted by the disruption of the MCT1/4-CAIX transport metabolon with small molecule inhibitors like BGal2C.

## 4. Materials and Methods

### 4.1. Beta-Galactose-2C Synthesis

The synthesis of BGal2C has been previously described by Murray et al. [47].

### 4.2. Carbonic Anhydrase Expression and Purification

CAII and CAIX-mimic were expressed in Escherichia coli BL21 pLysS (DE3) cells and purified as described in the literature [66,67]. Briefly, cells were grown in LB media, and protein expression was induced with 1 mM isopropyl β-D-1-thiogalactopyranoside and 1 mM ZnSO_4_ for 3 h [68]. Cell pellets were harvested with a centrifuge and lysed with a microfluidizer. The protein was loaded onto a p-Aminomethyl-benzenesulfonamide-Agarose column, and carbonic anhydrase was eluted with 0.4 M sodium azide. The carbonic anhydrase was buffer exchanged into 50 mM Tris 8.0 pH and concentrated to 10 mg/mL. Protein purity was checked with an SDS-PAGE gel for both CAII and CAIX.

### 4.3. Carbonic Anhydrase Crystallization

A 1:1 protein to precipitant solution (1.6 M sodium citrate, 50 mM Tris pH 7.8) in 5 µL was used with the hanging-drop vapor-diffusion method to set crystal trays of CAII and CAIX-mimic. The CA crystals were incubated at RT, and crystal growth was noted within a week. The CA crystals were soaked with BGal2C for 1 h. A 20% glycerol cryoprotectant solution was used to coat the CAII and CAIX crystals prior to flash-cooling with liquid nitrogen.

### 4.4. Carbonic Anhydrase X-Ray Crystallography Data Collection and Processing

X-ray crystallography diffraction data was collected at Stanford Synchrotron Radiation Lightsource (SSRL) with a EIGER detector. A crystal-to-detector distance of 150 mm and oscillation angle of 0.15° were used to collect data sets for a total of 1200 images. XDS was used to index and integrate diffraction data [69]. AIMLESS within the CCP4 program suite scaled the data into the P1 21 1 space group [70,71]. Molecular replacement determined the phases using PDB 3KS3 as a search model for the structure of CAII and PDB 4ZAO as a search model for the structure of CAIX-mimic [72,73]. Coot was used to modify the CA model and build in BGal2C. PHENIX performed refinements and generated restraint files for the ligand [74,75].

### 4.5. Molecular Modeling

The Ig1 domain of CD147 and catalytic domain of CAIX were docked and modeled as previously described in Ames et al. [12]. The PDBs used to generate the model were 3B5H for CD147 and 4ZAO for CAIX. The complex with BGal2C was generated by superposition of the CAIX-mimic BGal2C complex structure onto the Ames et al. CAIX structure. Figures were generated and analyzed in Pymol version 2.4 (Schrödinger, New York, NY, USA).

### 4.6. Measurements of Intracellular H^+^ Concentrations in Xenopus Oocytes

rMCT4 and hCAIX were expressed in *Xenopus* oocytes as previously described [76,77]. The procedure of oocyte removal from female *Xenopus* laevis frogs was approved by the Niedersächsisches Landesamt für Verbraucherschutz und Lebensmittelsicherheit, Oldenburg (33.19-42502-05-17A113).

Intracellular H^+^ concentrations were determined with ion-sensitive microelectrodes, as described in detail previously [77,78]. Oocytes were clamped to a holding potential of −40 mV. The measurements were carried out in oocyte saline (82.5 mM NaCl, 2.5 mM KCl, 1 mM CaCl_2_, 1 mM MgCl_2_, 1 mM Na_2_HPO_4_, 5 mM HEPES, pH 7.0) in the nominal absence of CO_2_/HCO_3_^−^ at RT. In lactate-containing solution, NaCl was replaced by equimolar amounts of Na-L-lactate. For CO_2_/HCO_3_^−^-buffered saline, NaCl was replaced by NaHCO_3_, and the solution was constantly aerated with 5% CO_2_/95% O_2_. The rate of change in intracellular H^+^ concentration was determined by linear regression fitting with OriginPro 8.6 (OriginLab Corporation, Northampton, MA, USA), as previously described [77].

### 4.7. Lactate Imaging in Single Cancer Cells

MDA-MB-231 cells and MCF-7 cells (DSMZ-No. ACC 732 and ACC 115) were purchased from the German Collection of Microorganisms and Cell Cultures (DSMZ, Braunschweig, Germany). Authentication of both cell lines was carried out by Eurofins Genomics Europe Applied Genomics GmbH, Ebersberg, Germany. Genetic characteristics were determined by PCR-single-locus-technology. A total of 16 independent PCR systems (D8S1179, D21S11, D7S820, CSF1PO, D3S1358, TH01, D13S317, D16S539, D2S1338, AMEL, D5S818, FGA, D19S433, vWA, TPOX and D18S51) were investigated using the Thermo Fisher (Waltham, MA, USA), AmpFlSTR^®^ Identifiler^®^ Plus PCR Amplification Kit. In parallel, positive and negative controls were carried out, yielding correct results. The cells tested negative regularly for contamination with mycoplasma.

Cells were cultured in RPMI-1640 medium with L-glutamine and NaHCO_3_ (R8758, Sigma-Aldrich, Schnelldorf, Germany) with L-glutamine and NaHCO_3_ and supplemented with 10% fetal bovine serum (F7524, Sigma-Aldrich) and 1% penicillin-streptomycin (P4333, Sigma). To trigger expression of CAIX, the cells were incubated under hypoxia (94% N_2_, 5% CO_2_, 1% O_2_) for 3–5 days. We have shown previously that 3–5 days under hypoxia is well tolerated by MDA-MB-231 and MCF-7 cells [12,48,49]. Relative changes in intracellular lactate concentration were measured using the lactate-sensitive FRET nanosensor Laconic, as previously described [12,48]. To determine the effect of BGal2C on lactate transport, cells were first superfused with lactate-containing saline in the absence of the inhibitor for 5 min (Figure 2B). After washout of lactate, the cells were superfused with saline containing 10 µM of BGal2C for 1 h. To prevent bleaching of the sensor, the recording was stopped during that time. After wash-in of the inhibitor, the cells were superfused with saline containing lactate and BGal2C. The rate of lactate uptake and release was determined by linear regression fitting using OriginPro 8.6. For the control, CAIX was knocked down using siRNA (Ambion Silencer^®^ Select anti CA9 siRNA, s2270, Life Technologies, Carlsbad, CA, USA), as previously described [48]. Knockdown efficiency was determined by qRT-PCR, as previously described [48]. At the RNA level, CAIX knockdown efficiency was 98.9% for MDA-MB-231 cells and 97.9% for MCF-7 cells.

### 4.8. In Situ Proximity Ligation Assay

Interaction between MCTs and CAIX in cultivated MDA-MB-231 and MCF-7 breast cancer cells was examined with the Duolink^®^ in situ Proximity Ligation Assay (PLA) Kit (DUO92103, Sigma-Aldrich). The protocol for the PLA has been previously described in detail [54]. The PLA was carried out with the following antibodies: goat anti-MCT1 polyclonal antibody (T-19), sc-14917, Santa Cruz Biotechnology (Dallas, TX, USA), 1 µg/mL; goat anti-MCT4 polyclonal antibody (C-17), sc-14930, Santa Cruz Biotechnology, 1 µg/mL; mouse anti-CAIX monoclonal antibody (M75), kindly provided by Dr. Silvia Pastorekova [79], 1 µg/mL. Actin filaments were stained with CruzFluor™ 488-labelled phalloidin (1:1000; sc-363791; Santa Cruz Biotechnology, Inc.). Nuclei were stained with 4′,6-diamidino-2-phenylindole (DAPI), added to the mounting medium (ProLong Gold Antifade Mountant; Thermo Fisher). Pictures were taken with a Leica SP5 confocal laser scanning microscope, using a 40× oil immersion objective. Specificity of the PLA was confirmed in a previous study by knockdown of CAIX [12]. To test the effect of BGal2C on the interaction between MCTs and CAIX, cells were incubated for 1 h in RPMI-1640 medium without serum, containing 10 µM of BGal2C. In control cells, BGal2C was omitted in the medium.

### 4.9. Western Blot Analysis

Protein expression in *Xenopus* oocytes was determined by western blot analysis, as previously described [55]. MCT4 was labelled with rabbit anti-rat MCT4 polyclonal antibody (AB3314P, Millipore, Burlington, MA, USA; 1:250). Specificity of the antibody was confirmed in native *Xenopus* oocytes. β -tubulin (anti-β-tubulin mouse monoclonal antibody, Sigma Aldrich; 1:4000) was used as a loading control. Primary antibodies were labeled with goat anti-rabbit or goat anti-mouse IgG horseradish peroxidase-conjugated secondary antibody (Santa Cruz Biotechnology Inc.; 1:4000). The chemiluminescence signal was detected with a ChemiDoc MP Imaging System (Bio-Rad, Hercules, CA, USA).

### 4.10. Calculation and Statistics

All data are shown as mean ± standard error of the mean. Significance in difference between paired samples was tested with a paired *t*-test. For unpaired groups of samples, significance in difference was calculated by One-Way ANOVA, followed by means comparison using Scheffé or Bonferroni test, depending on whether data sets show homogeneity of variance or not. Homogeneity of variance was tested with Levene’s test. All tests were performed with OriginPro 6.8. No outliers were removed from the data sets.

## Data Availability

All data are contained within the article or Supplementary Materials. The X-ray crystal structures of CAII and CAIX-mimic with BGal2C have been deposited to the PDB with accession code 7RRE and 7RRF, respectively.

## Supplementary Materials

The following supporting information can be downloaded at: https://www.mdpi.com/article/10.3390/ijms252211994/s1.
