# Supplementary material for: Disruption of the Physical Interaction Between Carbonic Anhydrase IX and the Monocarboxylate Transporter 4 Impacts Lactate Transport in Breast Cancer Cells
Source: Int J Mol Sci. 2024 Nov 8;25(22):11994. doi: 10.3390/ijms252211994 (PMC11593560; doi:10.3390/ijms252211994)
Supplement: Supplementary file 1 [file ijms-25-11994-s001.zip › ijms-3279299-supplementary.pdf]

Disruption of the physical interaction between carbonic anhydrase IX and the monocarboxylate transporter 4 impacts lactate transport in breast cancer cells

Jacob E. Combs[1+], Akilah B. Murray[1+], Carrie L. Lomelino[1], Mam Y. Mboge[1], Mario Mietzsch[1], Nicole A. Horenstein[2], Susan C. Frost[1], Robert McKenna[1*] and Holger M. Becker[3,4*]

[1]Department of Biochemistry and Molecular Biology, University of Florida, Gainesville, Florida, USA. [2]Department of Chemistry, University of Florida, Gainesville, Florida, USA. [3]Institute of Physiological Chemistry, University of Veterinary Medicine Hannover, Hannover, Germany. [4]Present address: Department of Gastroenterology, Hepatology, Infectious Diseases and Endocrinology, Hannover Medical School, Hannover, Germany. [+]Authors have contributed equally

*Corresponding authors: Robert McKenna (rmckenna@ufl.edu) and Holger M. Becker (Becker.Holger@mh-hannover.de)

Supplementary Information

Figure S1

Tables S1 and S2

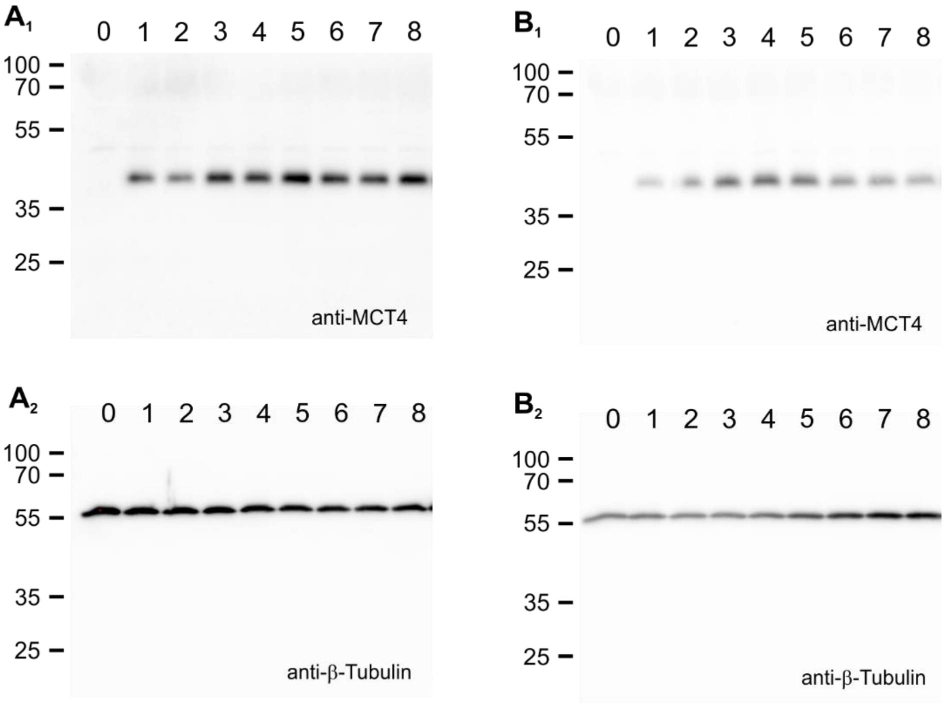

Figure S1: Effect of BGal2C on MCT4 expression in *Xenopus* oocytes. (**A**, **B**) Western blot against MCT4 (**A1**, **B1**) and β-Tubulin (**A2**, **B2**). 0: Native oocytes; 1: MCT4 expressing oocyte; 2: MCT4+CAIX coexpressing oocytes; 3: MCT4 expressing oocytes + 100 µM BGal2C (overnight incubation); 4: MCT4+CAIX coexpressing oocytes + 100 µM BGal2C (overnight incubation); 5: MCT4 expressing oocytes + 100 µM Galactose (overnight incubation); 6: MCT4+CAIX coexpressing oocytes + 100 µM Galactose (overnight incubation); 7: MCT4 expressing oocyte + 100 µM BGal2C (2h incubation time); 8: MCT4+CAIX coexpressing oocyte + 100 µM BGal2C (2h incubation time).

Table S1: X-ray crystallographic collection and refinement statistics of BGal2C bound to CAII.

Resolution range (Å)	32.14 - 1.44 (1.45 - 1.44)
Space group	$P2_1$
Unit cell *a,b,c* (Å) β (°)	42.4 41.4 72.0 104.3
Total reflections	138456 (8147)
Unique reflections	41691 (3072)
Multiplicity	3.3 (2.7)
Completeness (%)	95.3 (71.4)
Mean I/sigma(I)	23.3 (6.7)
Wilson B-factor	11.9
R-merge	0.03018 (0.0985)
R-meas	0.03589 (0.1219)
Reflections used in refinement	41691 (3072)
Reflections used for R-free	2118 (143)
R-work	0.1467 (0.1650)
R-free	0.1674 (0.1878)
Number of atoms (non-hydrogen, macromolecules, ligands, solvent)	2299, 2059, 58, 201
Protein residues	258
RMS (bonds) (Å)	0.009
RMS (angles) (Å)	1.11
Ramachandran favored (%)	97.66
Ramachandran allowed (%)	2.34
Ramachandran outliers (%)	0.00
Rotamer outliers (%)	0.45
Clashscore	2.4
B-factor (Å^2) average, macromolecules, ligands, solvent	14.8, 13,8, 28.6, 22.4

Statistics for the highest-resolution shell are shown in parentheses.

Table S2: X-ray crystallographic collection and refinement statistics of BGal2C bound to CAIX-mimic.

Resolution range (Å)	35.73 - 1.45 (1.50 - 1.45)
Space group	$P2_1$
Unit cell *a,b,c* (Å) β (°)	42.1 41.5 72.2,103.8
Total reflections	135323 (9539)
Unique reflections	39407 (3002)
Multiplicity	3.4 (3.2)
Completeness (%)	91.2 (69.9)
Mean I/sigma(I)	26.1 (9.0)
Wilson B-factor	12.0
R-merge	0.02779 (0.0879)
R-meas	0.03294 (0.1058)
Reflections used in refinement	39407 (3001)
Reflections used for R-free	1891 (156)
R-work	0.1529 (0.1817)
R-free	0.1693 (0.2300)
Number of atoms (non-hydrogen, macromolecules, ligands, solvent)	2336, 2059, 102, 213
Protein residues	257
RMS (bonds) (Å)	0.010
RMS (angles) (Å)	1.10
Ramachandran favored (%)	97.25
Ramachandran allowed (%)	2.75
Ramachandran outliers (%)	0.00
Rotamer outliers (%)	0.00
Clashscore	3.4
($Å^2$) average, macromolecules, ligands, solvent	16.4, 14.9, 38.0, 23.5

Statistics for the highest-resolution shell are shown in parentheses.
